# N-Acetyl-L-cysteine Protects the Enterocyte against Oxidative Damage by Modulation of Mitochondrial Function

**DOI:** 10.1155/2016/8364279

**Published:** 2016-11-27

**Authors:** Hao Xiao, Miaomiao Wu, Fangyuan Shao, Guiping Guan, Bo Huang, Bie Tan, Yulong Yin

**Affiliations:** ^1^Key Laboratory of Agro-Ecological Processes in Subtropical Region, Institute of Subtropical Agriculture, Chinese Academy of Sciences, Observation and Experiment Station of Animal Nutrition and Feed Science in South-Central China, Ministry of Agriculture, Hunan Provincial Engineering Research Center for Healthy Livestock and Poultry Production, Changsha, Hunan 410125, China; ^2^University of the Chinese Academy of Sciences, Beijing 10008, China; ^3^Department of Microbiology, Molecular Genetics, and Immunology, University of Kansas Medical Center, Kansas City, KS 66160, USA; ^4^Faculty of Health Sciences, University of Macau, Macau, Macau; ^5^College of Bioscience and Biotechnology, Hunan Agricultural University, Changsha, Hunan 410128, China; ^6^Hunan Collaborative Innovation Center for Utilization of Botanical Functional Ingredients, Changsha, Hunan 410000, China

## Abstract

The neonatal small intestine is susceptible to damage caused by oxidative stress. This study aimed to evaluate the protective role of antioxidant N-acetylcysteine (NAC) in intestinal epithelial cells against oxidative damage induced by H_2_O_2_. IPEC-J2 cells were cultured in DMEM-H with NAC and H_2_O_2_. After 2-day incubation, IPEC-J2 cells were collected for analysis of DNA synthesis, antioxidation capacity, mitochondrial respiration, and cell apoptosis. The results showed that H_2_O_2_ significantly decreased (*P* < 0.05) proliferation rate, mitochondrial respiration, and antioxidation capacity and increased cell apoptosis and the abundance of associated proteins, including cytochrome C, Bcl-XL, cleaved caspase-3, and total caspase-3. NAC supplementation remarkably increased (*P* < 0.05) proliferation rate, antioxidation capacity, and mitochondrial bioenergetics but decreased cell apoptosis. These findings indicate that NAC might rescue the intestinal injury induced by H_2_O_2_.

## 1. Introduction

The neonatal small intestine is particularly vulnerable to damage induced by endotoxin, and this damage may be involved in plasma and intracellular production of reactive oxygen species (ROS), resulting in cell apoptosis, reducing antioxidative capacity and mitochondrial dysfunction [1–3]. The intestinal epithelium, the border between the body and the environment, is the main place to transport the nutrient. And the enterocyte is the main target of harmful factors and stress, for example, toxin and ROS [4]. Moreover, a large of evidence suggests that oxidant derivatives and ROS are produced in excess by the inflamed mucosa and may be pathogenic factors in some intestinal diseases [5, 6]. Oxidative stress generated by an imbalance between ROS and antioxidants contributes to the pathogenesis of arthritis, cancer, cardiovascular, liver, and respiratory diseases [7]. ROS is generic and includes a wide variety of molecules, free radicals, or ions derived from molecular oxygen, for instance, singlet oxygen (O_2_), superoxide anion radical (O_2_^∙−^), hydrogen peroxide (H_2_O_2_), and hydroxyl radical (HO^∙^) [8]. ROS elicits a wide spectrum of responses [9]. Low doses of ROS are mitogenic and promote cell proliferation, while intermediate doses of ROS induce temporary or permanent growth arrest, and high doses of ROS cause cell death [9]. H_2_O_2_ is an abundant and stable form of ROS, responding to inflammation, cellular dysfunction, and apoptosis, which ultimately lead to tissue and organ damage. Mitochondrion is the main target of intracellular oxidative stress and is regarded as the main source for endogenous ROS. Previous studies showed that an acute, noncytotoxic dose of H_2_O_2_ caused a delay fragmentation of the mitochondrial reticulum and depressed the mitochondrial membrane potential and maximal respiratory rate [10]. Therefore, H_2_O_2_-induced damage is a reproducible and simple model to cause oxidative stress.

N-Acetylcysteine (NAC), the precursor of L-cysteine, is known as an antioxidant that acts as a source of thiols and functions in glutathione synthesis, glutathione peroxidase (GPx) activity, and detoxification and acts directly on reactive oxidant radicals as a superoxide scavenger which interacts with ROS such as HO^∙^ and H_2_O_2_ [7]. The previous study showed that weaning increased the concentrations of NO and H_2_O_2_ in the serum in postweaning piglets, and feeding antioxidant-containing diets could prevent the ROS-induced damage and suppress oxidative stress [11]. There is growing evidence that NAC might be a promising agent to improve intestinal health in piglets [12]. NAC supplementation could alleviate the mucosal damage and improve the absorptive function of the small intestine in lipopolysaccharide- (LPS-) challenged piglets [13]. NAC regulates antioxidative responses, cell apoptosis, and epidermal growth factor gene expression under acetic acid challenges [6]. However, the mechanisms by which NAC exerts protective effects in intestinal damage are incompletely understood.

We hypothesize that NAC enhances cell growth and mitochondrial bioenergetics and decreases cell apoptosis on H_2_O_2_-induced oxidative damage in intestinal cells. The present study was designed to test this hypothesis using a model of H_2_O_2_-induced damage of intestinal porcine epithelial cells (IPEC-J2).

## 2. Materials and Methods

### 2.1. Cell Culture

The reagents and cell culture refer to our previous study [14]. High-glucose (25 mM) Dulbecco's modified Eagle's (DMEM-H), fetal bovine serum (FBS), and antibiotics were procured from Invitrogen (Grand Island, NY, USA). Plastic culture plates were manufactured by Corning Inc. (Corning, NY, USA). Unless indicated, all other chemicals were purchased from Sigma-Aldrich (St. Louis, MO, USA).

IPEC-J2 cells were seeded and cultured with DMEM-H medium containing 10% FBS, 5 mM l-glutamine, 100 U/mL penicillin, and 100 *μ*g/mL streptomycin at 37°C in a 5% CO_2_ incubator. After an overnight incubation, the cells were changed to culture in basal medium containing 0 or 800 *μ*M NAC. The following day, 0 or 100 *μ*M H_2_O_2_ was added for 4 hours and then the mediums were changed as before. The cells were collected for further research after 2-day incubation.

### 2.2. Cell Viability Assay

About 1 × 10^4^ cells per well of IPEC-J2 cells were seeded in 96-well plates and grown as usual. After incubation in 0, 500, 650, 800, or 1000 *μ*M NAC medium for 24 h, then 100 *μ*M H_2_O_2_ were added for 4 h. The wells were washed and fresh basal medium was replaced. Cell Counting Kit-8 (CCK-8) was added to each well, incubated for 2 h, and read on the spectrophotometer at 450 nm; the measured absorbance is proportional to the number of viable cells.

### 2.3. DNA Synthesis Measurement

IPEC-J2 cells (1 × 10^4^) were seeded in 96-well plates and cultured for a 2-day period. DNA synthesis during cell proliferation in all treatment groups was quantified using 5-ethynyl-2′-deoxyuridine (EdU; Invitrogen) incorporation using Cell-Light EdU Kit (Rui Bo Biotechnology Limited Company, Guangzhou, China), as described in our previous studies [1]. Briefly, IPEC-J2 cells were cultured in DMEM-H mediums containing 50 *μ*M EdU for 1 h. An Olympus BX51 microscope (Olympus, Japan) was used to observe EdU-positive cells. Images of the Apoll® 567 Hoechst 33342 were captured. The percentage of EdU-positive cells was expressed as the ration of red nuclei cells to blue nuclei cells in at least five different microscopic fields randomly selected for counting at 200-fold magnification.

### 2.4. Detection of Antioxidation Capacity

IPEC-J2 cells (50 × 10^4^) were seeded in 10 cm dishes for determination of total antioxidant capacity (T-AOC) and lactate dehydrogenase (LDH) using their corresponding assay kits (Nanjing Jiancheng, Nanjing, China) according to the manufacturer's instructions [2]. All samples were measured by UV/visible spectrophotometer-UV-2450 (SHIMADZU, Kyoto, Japan) to get the results.

### 2.5. Flow Cytometry Analysis

IPEC-J2 cells (10 × 10^4^) were seeded in 6-well cell culture plates for flow cytometry analysis. After a 2-day period of culture in DMEM-H medium containing 0 or 800 *μ*M NAC and 0 or 100 *μ*M H_2_O_2_, medium and cells were collected separately. About 1 × 10^6^ cells were pelleted at 16 000 ×g for 5 min. The supernatant was removed and 1 mL of 70% cold ethanol was slowly added during vigorous mixing. Samples were stored at 4°C. Cells were washed once with ice-cold PBS and resuspended in 1 mL of staining reagent containing 50 mg/mL PI and 100 mg/mL RNase for 30 min in the dark. To assess apoptosis, harvested cells were stained with PI/Annexin-V-FITC (KeyGEN, Nanjing, China) according to the manufacturer's instructions. Cell cycle arrest and apoptosis were analyzed by flow cytometry (BD FACSCalibur, USA). Fluorescence of PI and Annexin-V-FITC was monitored at 630 nm and 525 nm, respectively.

### 2.6. Metabolic Assays

The XF-24 Extracellular Flux Analyzer and Cell Mito Stress Test Kit from Seahorse Biosciences were used to examine the effects of NAC treatment on mitochondrial respiration in H_2_O_2_-induced cells as described by Tan et al. [15]. After a 2-day period of culture, the basal medium was changed prior to the bioenergetic measurements to serum-free unbuffered (without sodium bicarbonate) DMEM medium base supplemented with 2 mM L-glutamine, 25 mM D-glucose, and 1 mM sodium pyruvate, at pH 7.4 ± 0.1 at 37°C. To measure indices of mitochondrial function, oligomycin, carbonyl cyanide-p-trifluoromethoxyphenylhydrazone (FCCP), and rotenone and antimycin A were injected sequentially at the final concentrations of 0.5, 1, and 1 *μ*M, respectively. This allowed for an estimation of the contribution of non-ATP–linked oxygen consumption (proton leak) and ATP-linked mitochondrial oxygen consumption (ATP production). The maximal respiration capacity was determined using the FCCP-stimulated rate. The spare respiratory capacity was represented by the maximal respiratory capacity subtracted from the baseline oxygen consumption rate (OCR). The residual oxygen consumption that occurred after addition of rotenone and antimycin A was ascribed to nonmitochondrial respiration and was subtracted from all measured values in the analysis [1]. Owing to the effects of NAC on IPEC-J2 proliferation, total cellular protein was determined and used to normalize mitochondrial respiration rates.

### 2.7. Detection of TCA Cycle Intermediates by GC-MS

IPEC-J2 cells (50 × 10^4^) were seeded in 10 cm dishes for GC-MS analysis as described by Morita et al. [16]. Briefly, cells were washed with PBS and treated by 0.25% trypsin. And then cells were collected and pelleted at 1000 ×g for 5 min. After being quenched using 500 *μ*L of prechilled 50% (v/v) methanol, cells were centrifuged at 1000 ×g for 5 min and then removed and added 500 *μ*L of prechilled 100% (v/v) methanol. Cells were measured by an Agilent 7890B-5977A GC-MS equipped with HP-5ms (30 m × 250 *μ*m × 0.25 *μ*m) capillary column (Agilent J&W, Santa Clara, CA, USA). All metabolites were previously validated using authentic standards (Sigma).

### 2.8. Western Blotting Analysis

Cells were rinsed twice using PBS, harvested, pelleted by centrifugation, and lysed in RIPA buffer (150 mM NaCl, 1% Triton X-100, 0.5% sodium deoxycholate, 0.1% SDS, 50 mM Tris-HCl at PH 7.4), plus a protease inhibitor cocktail and phosphatase inhibitors. Protein concentrations of cell homogenates were measured using the BCA method and bovine serum albumin as standard, as described by our previous studies [1]. All samples were adjusted to an equal concentration. Soluble proteins were subjected to SDS-PAGE and transferred to PVDF membranes, blocked with 5% nonfat milk in TBS-with 0.05% Tween-20 for 1 h, and incubated overnight with the following primary antibodies overnight at 4°C with gentle rocking: cytochrome C (1 : 1,000; Cell Signaling Technology), Bax (1 : 1,000; Cell Signaling Technology), caspase-3 (1 : 1,000; Cell Signaling Technology), Bcl-XL (1 : 400; Santa Cruz Biotechnology, Dallas, TX), cleaved caspase-3 (1 : 400; Santa Cruz Biotechnology, Dallas, TX), or *β*-actin (1 : 400; Santa Cruz Biotechnology, Dallas, TX), followed by horseradish peroxidase-linked secondary antibodies. The protein bands were visualized using a chemiluminescent reagent. The density of the protein bands was determined using the Alpha Imager 2200 software (Alpha Innotech Corporation) and normalized the data with inner control.

### 2.9. Statistical Analysis

Results are expressed as mean ± SEM. The statistical analysis was performed by one-way ANOVA using SPSS 17.0 (SPSS Inc., Chicago, IL, USA). Probability values < 0.05 were considered statistically significant.

## 3. Results

### 3.1. Effects of H_2_O_2_ and NAC on the Cell Viability of IPEC-J2 Cells

**Figure 1:**
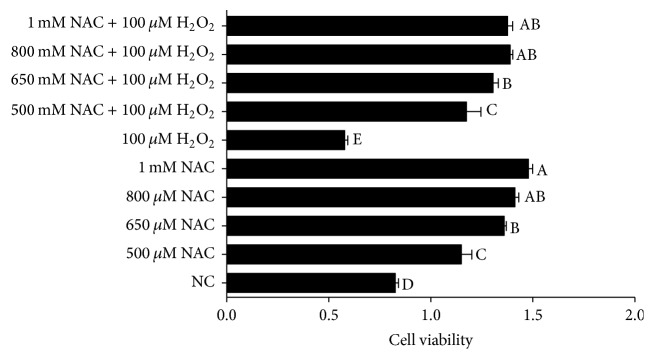
Cell proliferation in IPEC-J2 cells. Cells were treated with 0 (NC) to 1000 *μ*M NAC and 0 or 100 *μ*M H_2_O_2_, respectively, for a 2-day period. Cell viability was quantified by CCK-8 assay. Data are expressed as means ± SEM of at least three independent experiments. ^a–e^Values with different letters are significantly different (*P* < 0.05).

**Figure 2:**
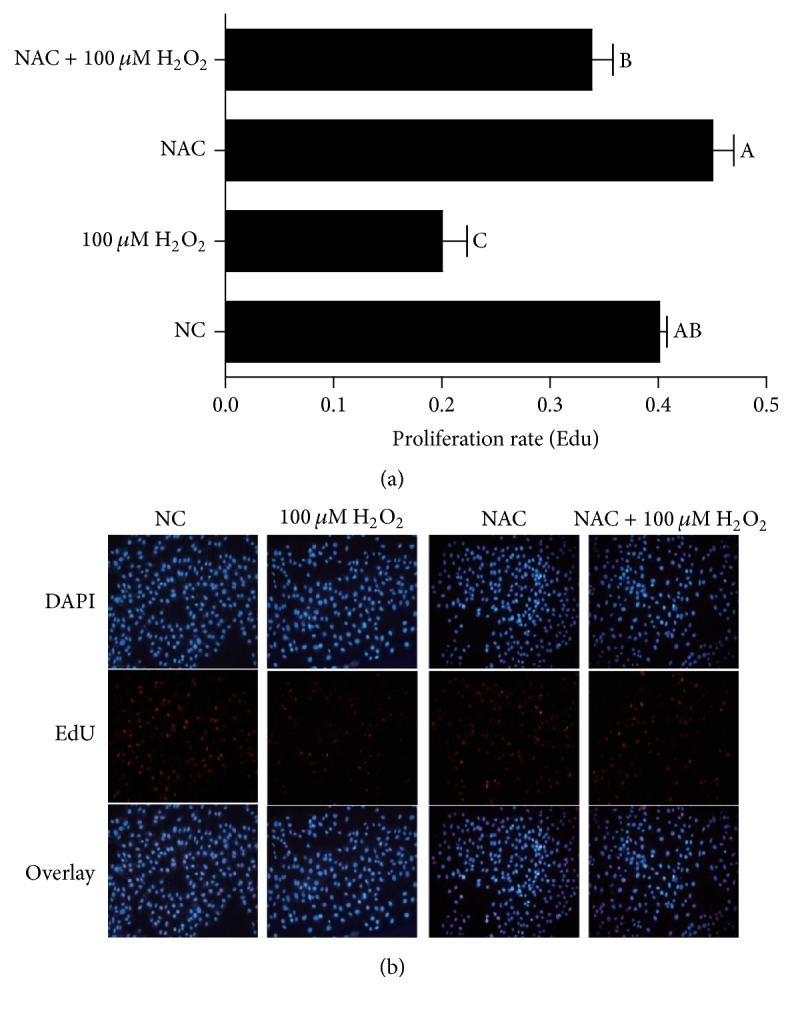
DNA synthesis in IPEC-J2 cells. DNA synthesis during the proliferation of IPEC-J2 cells was quantified by EdU incorporation (red color) using Cell-Light™ EdU Kit (Rui Bo Biotechnology Limited Company, Guangzhou, China). Nuclei are shown in blue color. Cells were treated with 0 (NC) or 800 *μ*M NAC and 0 or 100 *μ*M H_2_O_2_, respectively. (a) The percentage of EdU-positive cells (the number of red nuclei versus the number of blue nuclei in at least five different microscopic fields of vision). (b) Representative images of EdU staining (magnification ×200) of cells. Data are expressed as means ± SEM of at least three independent experiments. ^a–c^Values with different letters are significantly different (*P* < 0.05).

Viability assay of IPEC-J2 cells was performed by firstly treating the cells with different concentrations of NAC (0, 500, 650, 800, and 1000 *μ*M, resp.) for one day and then with 100 *μ*M H_2_O_2_ for 4 h. The results indicated that 100 *μ*M H_2_O_2_ decreased IPEC-J2 cell viability, while addition of NAC enhanced cell viability of H_2_O_2_-treated IPEC-J2 cells in a dose-dependent manner, and 800 and 1000 *μ*M NAC addition showed the best promotion effects compared with the 0 and 500 *μ*M NAC treatment in H_2_O_2_-treated cells (*P* < 0.05) (Figure 1). The results of EdU incorporation illustrated in Figure 2 have showed that the percentages of EdU-positive cells were significantly decreased in response to H_2_O_2_ treatment (*P* < 0.05), while addition of NAC to cells showed a tendency to increase the percentages of EdU-positive cells compared with NC group.

### 3.2. Mitochondrial Bioenergetics

**Figure 3:**
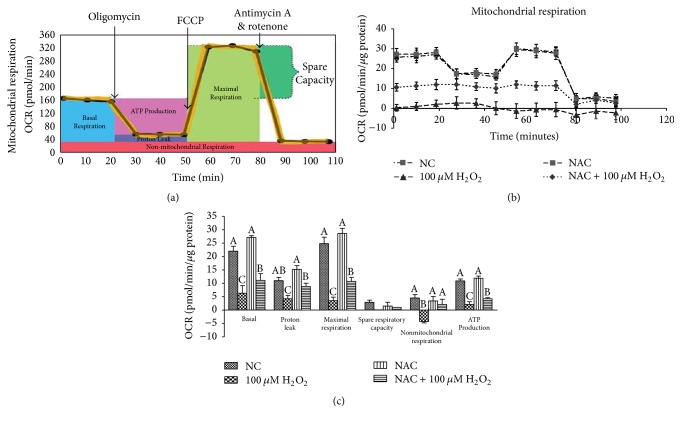
Mitochondrial respiration of IPEC-J2 cells measured by the XF-24 Extracellular Flux Analyzer and Cell Mito Stress Test Kit from Seahorse Biosciences (North Billerica, MA, USA). (a) Schematic and (b) oxygen consumption rate (OCR) assessed by extracellular flux analysis. OCR was measured under basal conditions followed by the sequential addition of oligomycin (0.5 *μ*M), FCCP (1 *μ*M), rotenone (1 *μ*M), or antimycin A (1 *μ*M). Each data point represents an OCR measurement. (c) Individual parameters for basal respiration, proton leak, maximal respiration, spare respiratory capacity, nonmitochondrial respiration, and ATP production were determined. Cells were treated with 0 (NC) or 800 *μ*M NAC and 0 or 100 *μ*M H_2_O_2_, respectively. Data were expressed as means ± SEM of at least three independent experiments. ^a–c^Values with different letters are significantly different (*P* < 0.05).

The results of mitochondrial respiration in IPEC-J2 cells are shown in Figure 3. Addition of 100 *μ*M H_2_O_2_ gradually decreased (*P* < 0.05) individual parameters for basal respiration, proton leak, maximal respiration, nonmitochondrial respiration, and ATP production in cells while addition of NAC elevated the rate of mitochondrial respiration in 100 *μ*M H_2_O_2_-treated cells (*P* < 0.05) but not in normal cells.

### 3.3. TCA Cycle Intermediates

**Figure 4:**
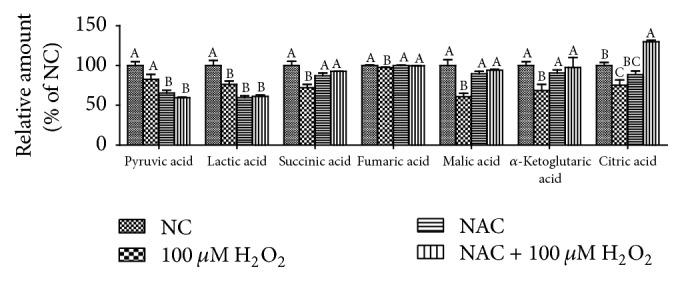
The TCA cycle intermediates, pyruvic acid, and lactic acid of IPEC-J2 cells measured by an Agilent 7890B-5977A GC-MS equipped with HP-5ms (30 m × 250 *μ*m × 0.25 *μ*m) capillary column (Agilent J&W, Santa Clara, CA, USA). Cells were treated with 0 (NC) or 800 *μ*M NAC and 0 or 100 *μ*M H_2_O_2_, respectively. Data were expressed as means ± SEM of at least three independent experiments. ^a–c^Values with different letters are significantly different (*P* < 0.05).

The relative content of pyruvic acid, lactic acid, and TCA cycle intermediates (citric acid, alpha-ketoglutarate, succinic acid, fumaric acid, and malic acid) of IPEC-J2 cells are illustrated in Figure 4. Addition of 100 *μ*M H_2_O_2_ significantly decreased lactic acid, and TCA cycle intermediates compared with the NC treatment (*P* < 0.05). Compared to NC treatment, addition of 800 *μ*M NAC significantly decreased the content of pyruvic acid and lactic acid (*P* < 0.05), while there were no differences in the contents of pyruvic acid, lactic acid, succinic acid, fumaric acid, malic acid and *α*-ketoglutaric acid between NAC and NAC + H_2_O_2_ treatments (*P* > 0.05).

### 3.4. Antioxidative Capacity

**Figure 5:**
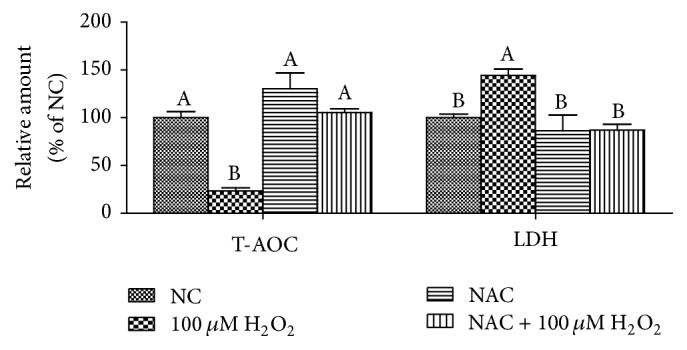
The concentrations of T-AOC and LDH in the IPEC-J2 cells. Cells were treated with 0 (NC) or 800 *μ*M NAC and 0 or 100 *μ*M H_2_O_2_, respectively. Data were expressed as means ± SEM of at least three independent experiments. ^a–b^Values with different letters are significantly different (*P* < 0.05).

The concentrations of T-AOC and LDH are presented in Figure 5. Compared with the NC group, 100 *μ*M H_2_O_2_ significantly decreased the concentrations of T-AOC but increased the concentrations of LDH (*P* < 0.05). However, in H_2_O_2_-treated cells, addition of NAC markedly increased the concentrations of T-AOC and decreased LDH leakage into the culture medium (*P* < 0.05).

### 3.5. Cell Apoptosis

**Figure 6:**
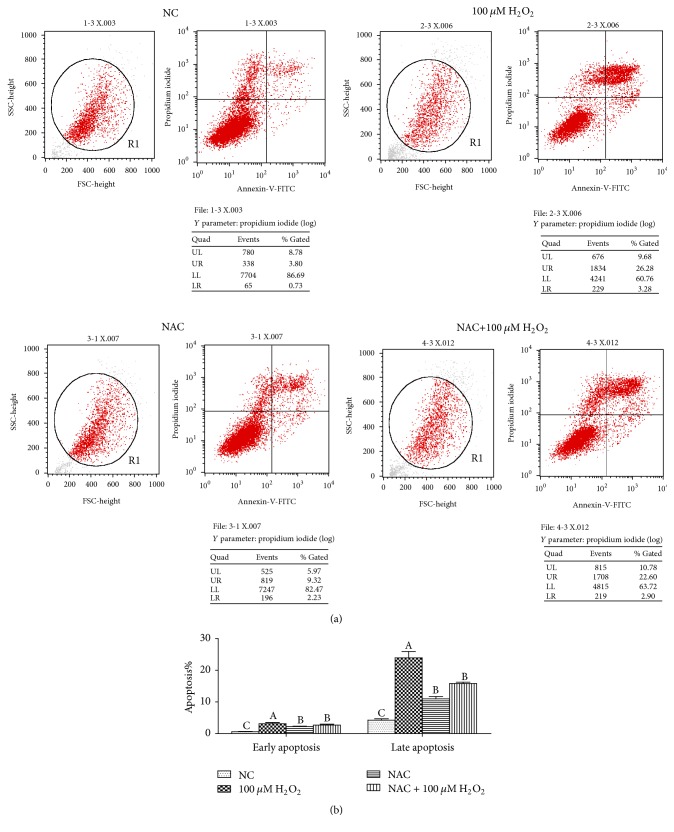
Cell apoptosis in the IPEC-J2 cells. (a) Representative flow cytometry diagrams and (b) apoptosis rate. Cells were treated with 0 (NC) or 800 *μ*M NAC and 0 or 100 *μ*M H_2_O_2_, respectively. Data were expressed as means ± SEM of at least three independent experiments. ^a–c^Values with different letters are significantly different (*P* < 0.05).

Cell apoptosis was analyzed by Annexin-V-FITC/PI staining, the results showed that compared with the NC group, 100 *μ*M H_2_O_2_ significantly increased the percentage of both early and late apoptosis of cells, and 800 *μ*M NAC addition also increased the apoptosis rate (*P* < 0.05). However, in H_2_O_2_-treated cells, addition of 800 *μ*M NAC significantly decreased the percentage of early and late apoptosis (*P* < 0.05) (Figure 6).

### 3.6. The Relative Protein Expression Levels of Cell Apoptosis

**Figure 7:**
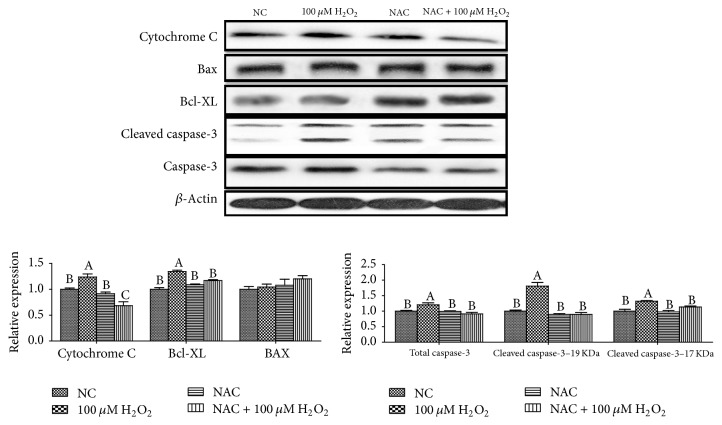
Abundances of proteins (cytochrome C, Bax, Bcl-XL, cleaved caspase-3, and caspase-3) in IPEC-J2 cells determined by western blot analysis. Cells were treated with 0 (NC) or 800 *μ*M NAC and 0 or 100 *μ*M H_2_O_2_, respectively. Data were expressed as means ± SEM of at least three independent experiments. ^a–c^Values with different letters are significantly different (*P* < 0.05).

The relative expression levels of cytochrome C, Bax, B-cell lymphoma/leukaemia-XL (Bcl-XL), cleaved caspase-3, and total caspase-3 proteins are shown in Figure 7. Addition of 100 *μ*M H_2_O_2_ significantly increased protein levels for cytochrome C, Bcl-XL, cleaved caspase-3, and total caspase-3 proteins (*P* < 0.05), while addition of 800 *μ*M NAC significantly decreased the above parameters in 100 *μ*M H_2_O_2_-treated cells (*P* < 0.05).

## 4. Discussion

NAC has promising effects in different diseases, including cancer, liver toxicity, cardiovascular diseases, and metal toxicity [17], due to its role in attenuating pathophysiological processes including oxidative stress, apoptosis, and mitochondrial dysfunction [18]. In addition, NAC could attenuate inflammation in the liver of LPS-treated mice [19]. Similarly, supplement of NAC has been reported to improve growth performance and energy status, reduce inflammation, and ameliorate tissue damage [12]. Recently, Yi et al. have found that NAC could stimulate protein synthesis and inhibit proteolysis in IPEC-1 cells [20]. In the present study, we found that NAC could not only ameliorate H_2_O_2_-induced cell growth inhibition, but also attenuate mitochondrial dysfunction in H_2_O_2_-treated cells. Furthermore, NAC downregulated the mitochondria-depended apoptosis in H_2_O_2_-treated cells. Therefore, our data suggest that NAC might repair intestinal damage through improving the mitochondrial function.

Intragastric or intraperitoneal administration of H_2_O_2_ could decrease growth performance and caused oxidative stress [21–23]. Furthermore, previous studies showed that addition of H_2_O_2_ (300 *μ*M) to chicken intestinal epithelial cells for 24 h significantly decreased cell survival and SOD activity [24]. Similarly, the results indicated that H_2_O_2_ at 100 *μ*M for 4 h decreased the growth of IPEC-J2. Additionally, the percentages of EdU-positive cells were decreased in response to 100 *μ*M H_2_O_2_ treatment. In addition, the reports show that excess intracellular ROS level could cause oxidative damage to lipids, DNA, and proteins via apoptosis [25]. In the present study, mitochondrial function was destroyed in H_2_O_2_ treatment, which is in accordance with results from Fan et al. [10]. These data indicate that H_2_O_2_ induces mitochondrial ROS production and then leads to DNA damage in IPEC-J2 cells. The report by Yi et al. indicated that NAC increased the growth of IPEC-1 cells and suggested that NAC at low concentrations (<1 mM) could stimulate cell growth [20]. In line with these results, this experiment showed that addition of NAC at 500–1000 *μ*M to 100 *μ*M H_2_O_2_ treatment increased the cell viability. And adding NAC at 800 *μ*M to 100 *μ*M H_2_O_2_ increased the cell proliferation, indicating that NAC might improve the H_2_O_2_-induced cell growth damage.

There is increasing evidence supporting that NAC improve redox status and directly react with oxidative metabolites [12, 26, 27]. NAC protects cells against oxidative stress through reducing glutathione (GSH) and interacting with ROS [12]. In this study, H_2_O_2_ exposure induced oxidative stress evidenced by decreased cell viability, inhibited T-AOC, and increased leakage of LDH, while NAC treatment markedly improved antioxidant system. Mitochondria are the powerhouses of the cell, producing a considerable share of cellular ATP and playing a central role in cellular function and metabolism [28]. Our previous report showed that mitochondrial dysfunction was observed with decrease in the basal respiration, maximal respiration, and nonmitochondrial respiration after LPS treatment [1]. The present data also demonstrated that mitochondrial function damage induced by H_2_O_2_ was observed, showing decrease in basal respiration, proton leak, maximal respiration, spare respiratory capacity, nonmitochondrial respiration, and ATP production. In vivo, NAC has been determined to improve mitochondrial uncoupling and respiration in inflamed intestines [26]. Our results showed that NAC could improve mitochondrial bioenergetics in H_2_O_2_-treated cells. The previous studies have reported that NAC improved oxygen delivery [29] and systemic oxygen consumption [30] and regulated mitochondrial TCA cycle metabolism by stimulation of carbon flux through pyruvate dehydrogenase, a key enzyme for hepatocellular mitochondrial energy metabolism by acetyl-CoA supply [31, 32]. Our results were in agreement with these previous ideas. The results showed that NAC influenced metabolism of cellular pyruvic acid, lactic acid, succinic acid, malic acid, and citric acid, which contribute to mitochondrial redox balancing and are transported into mitochondria to affect ATP production by oxidative phosphorylation [33, 34].

Reports showed that ROS and mitochondrial dysfunction could mediate apoptosis, indicating that ROS are important in cellular apoptosis [35]. Based on studies from various cell types, it is increasingly clear that NAC could inhibit the cell apoptosis [12, 36, 37]. Flow cytometry analysis showed that 100 *μ*M H_2_O_2_ significantly induced cell apoptosis, while NAC could attenuate this effect of H_2_O_2_ by inhibiting cell apoptosis at both early and late stages, which is consistent with Mayer and Noble and Shen et al.'s studies [38, 39]. Unbelievably, this inhibition of NAC on cell apoptosis is only observed in H_2_O_2_ treated cells and NAC induced cell apoptosis in normal cells, which require further research. Cytochrome c is released from mitochondria due to formation of a channel, the mitochondrial apoptosis-induced channel, in the outer mitochondrial membrane, and serves a regulatory function as it precedes morphological change associated with apoptosis. The report showed that apoptosis was mediated via the intrinsic pathway. Loss of mitochondrial membrane potential increased release of cytochrome C in cytosol and activated some proapoptotic molecules (Bax, cleaved caspase-9, caspase-3, and so on) and caused downregulation of Bcl-2 which happened in a dose-dependent manner [40]. The previous study has found that supplementation with NAC attenuated caspase-3 protein expression in the small intestine of LPS-challenged pigs [13]. In the current study, we noted that the relative proteins expressions of cell apoptosis were elevated in IPEC-J2 cells after H_2_O_2_ treatment but were reduced when NAC was added into IPEC-J2 cells pretreated with H_2_O_2_. Thus, the beneficial effects of NAC may be associated with attenuating cell apoptosis.

In summary, H_2_O_2_ induced mitochondrial dysfunction and cell apoptosis, while NAC promoted DNA synthesis, mitochondrial bioenergetics, and mitochondria-depended apoptosis in intestinal epithelial cells. Possible mechanisms for the cytoprotective effect of NAC on H_2_O_2_-induced damage in IPEC-J2 cells scavenged the H_2_O_2_ and then improved cell proliferation, TCA cycle, and mitochondria function and reduced cell apoptosis and death. Results from these studies have important implications for the use of NAC in the clinical management of oxidative damage in the neonatal pigs.
